# A dataset of governments’ economic responses to COVID-19

**DOI:** 10.1016/j.dib.2023.109021

**Published:** 2023-02-27

**Authors:** Simon Porcher

**Affiliations:** Université Paris Panthéon-Assas, LARGEPA, 1 rue Guy de La Brosse, 75005 Paris, France

**Keywords:** Fiscal policies, COVID-19, Economic measures, Panel data

## Abstract

We introduce a new dataset of ten economic measures in percentage of gross domestic product implemented by governments worldwide between January 2020 and June 2021 to fight COVID-19. The measures coded include fiscal measures (wage support, cash transfers, in-kind transfers, tax cuts, sectorial support and credit schemes), tax deferrals, off-budget measures, and main policy rate cuts. The data can be used to study the impact of economic measures on different outcomes, and to understand the diffusion of economic policies during crises.

**Specifications Table**

**Table:** 

Subject	Economics
Specific subject area	Macroeconomics
Type of data	Stata
How the data were acquired	The data were collected via a wide variety of sources. The two main sources are the IMF fiscal monitor database in response to the COVID-19 pandemic and the IMF COVID-19 policy tracker. Secondary sources include the draft budgetary plans of the European Union countries published on the website of the European Commission, the Organization for Economic Cooperation and Development (OECD), the announcements of central governments and ministries, and official press articles. These secondary sources were useful in obtaining the exact date of the implementation of policies. We cross-checked the information to ensure its accuracy and adapted the figures when necessary. All other sources than the IMF are listed in an Excel file (“Source.xls”) on the repository.
Data format	Analyzed and aggregated.
Description of data collection	The dataset is based on the manual recording of economic measures implemented all around the world. Three different coders worked from January 2021 until June 2021 on the coding. The coding script was cross-checked by each coder on several occasions and several different points in time. This systematic verification process ensures a homogeneous coding method and avoids mistakes, especially those linked to contradictory interpretations of the classification of the different measures. It also allows, when necessary, correction of the script over time in cases where previously announced measures turned out to be misestimated or unimplemented. We used the IMF Fiscal Monitor database and the IMF COVID-19 Policy Tracker to obtain information about the amounts spent. When necessary, we also used alternative sources to more precisely categorize the expenditures. All the extra sources that were used to feed the code are reported in an Excel file, which is available on the repository of the dataset. This ensures transparent, impartial and consistent coding. These sources, however, had to be consistent with the information supplied by the IMF. We are aware that collecting data from other sources might lead to the importation of any measurement issues they bring with them. However, we did our best to triangulate the information from the IMF with other sources. The raw data described in this article is merged with the United Nations Geoscheme's list of continents to produce descriptive statistics at the continent level.
Data source location	Primary data sources: IMF Fiscal policies Database: Fiscal Policies Database (imf.org) IMF Policy responses to COVID-19: https://www.imf.org/en/Topics/imf-and-covid19/Policy-Responses-to-COVID-19 OECD Policy Responses to Coronavirus: OECD Policy Responses to Coronavirus (COVID-19) EU Draft Budgetary Plan: Draft budgetary plans 2021 (europa.eu) Secondary data sources: United Nations Georeference Scheme: UNSD — Methodology
Data accessibility	Repository name: GitHub Data identification number: Direct URL to data: simonporcher/Fiscal_Response2covid19: Dataset of governments' fiscal and off-budget measures to respond to COVID-19 (github.com) https://doi.org/10.5281/zenodo.7504461

## Value of the Data

• This paper introduces a new dataset collecting information about the economic measures implemented by governments to fight COVID-19.
• The dataset is useful to inform citizen, policy-makers, and the media about the breakdown of governments’ spending during COVID-19.
• Further research could use this dataset to assess the impact of various measures on economic growth, or aggregated measures of stress or mental health, across the world.
• The dataset could be used to study the diffusion of economic policies around the world.

## 1 Objective

Several research groups expressed the intention to inventory all the measures taken by governments during the COVID-19 pandemic [1,2,3]. However, most of these efforts have primarily focused on NPIs and have rarely included the systematically quantified expenses related to a variety of economic measures. Some projects either focus on some specific measures or on some geographical areas. For example, Gentilini et al. [4] created a database that lists social protection actions taken by governments during the COVID-19 pandemic in 151 countries but does not cover overall fiscal responses. Other projects have focused on geographical areas, such as the Asian Development Bank's COVID-19 Policy or the COVID-19 Observatory in Latin America and the Caribbean.^1^ In this paper, we introduce a new dataset of government COVID-19 fiscal responses [5]. It intends to categorize government spending directed at COVID-19 for each country worldwide as precisely as possible. It contains both on-budget measures (targeted at individuals, workers or firms) and off-budget measures (including credit schemes and tax deferrals). By including a quantitative dimension of the measures taken in the form of amounts spent for different measures, the database adds much value to the study of the types of policy instruments used as well as their outcomes following implementation during the pandemic.

## 2 Data Description

Based on our reading of the economic measures, we identified 10 standard economic measures that have been taken by governments. For each country-day, we coded the percentage of 2019 GDP spent by the government on different economic measures, usually following a fiscal plan. Coding in percentage, rather than in amounts, allows us to make relevant cross-country comparisons. We used the 2019 GDP as a basis for comparison because countries have been affected very differently by the pandemic. As it is impossible to compute the exact fluxes of government spending on a given day, we coded the percentage disbursed on a given measure at the stated starting day of the plan. When this information could not be retrieved, we used the day of the bill's passage. To capture the real effort of governments, we only considered those amounts that have been disbursed. As governments can announce policies that are sometimes not funded, particularly in developing countries that sometimes rely on foreign or international financial institutions to fund the announced measures, our dataset captures only those measures that were both implemented and funded by governments.

The coded variables are as follows:

- *fiscal*. This is the global amount spent on a given date, which is then divided between various categories. Fiscal includes additional or foregone revenues that had been specifically targeted at dealing with COVID-19. It does not account for delayed revenues such as tax deferrals and off-budget measures that do not directly affect the annual fiscal deficit. The breakdown of *fiscal* includes *wage support, cash transfers, in-kind transfers, sectoral support, tax cuts*, and *credit schemes*.
- *wage* gathers all the measures related to wage replacement for COVID-19-related unemployment, subsidies for job retention schemes, short-time work schemes, bonuses for some workers and increases in pensions. *Wage support* is conditional on employment status, e.g., being employed, retired, or recently unemployed.
- *cash* gathers all cash transfers that target individuals rather than jobs. These transfers can either be universal, specifically targeted at some vulnerable populations including elderly individuals, students, and single mothers, or conditional on income. Cash transfers can be distributed in various ways: Countries can digitally transfer money to the individuals, hand cash or cheques at the post office or the bank, or provide individuals with ATM cards to withdraw money.
- *inkind* includes all in-kind benefits provided to the population. It usually takes the form of food baskets or food vouchers delivered to the population, especially to vulnerable households. In-kind transfers also include free public housing rents or electricity bills or vouchers for holidays. We do not include free vaccines or health vouchers in this measure. All spending related to health are integrated in the sectoral support.
- *sectorial* gathers the measures targeted at companies, administrations, or governments facing difficulties because of the pandemic. In some cases, they are sectorial and intended to protect strategic affected sectors, such as tourism, airlines, or culture-related firms. In other cases, the government may seek to support local governments or the health sector, e.g., to support vaccination efforts. We group in this variable all measures that are targeted at a given sector. This measure captures fiscal spending that is targeted at institutions rather than at individuals.
- *taxc* is the amount of foregone tax revenues related to tax cuts. It covers all types of taxes, from value-added to income taxes for individuals and corporations.
- *creditin* are guarantees and lines of credits opened by the government that are included in the annual budget to counteract the effects of COVID-19 on both firms and individuals. In most countries, credit schemes are targeted at firms rather than individuals.
- *taxd* is the amount of delayed taxes. Tax deferrals do not lead to foregone revenues, as the exempted individuals will have to pay taxes at a later date.
- *offbudget* comprises below the line measures, such as equity injections, asset purchases, or loans, and other contingent liabilities, such as off-budget state guarantees on loans and the activity of public corporations on behalf of the government. Equity injections can be directed toward affected companies such as airlines or railway services. Many countries also created off-budget funds guaranteed by the State to provide quasi-equity support to firms. National development banks have also run quasi-fiscal operations on behalf of the State.
- *rate* is meant to capture the evolution of policy rates set up by the Central Bank. The values taken by the variable translate the nominal evolution. For example, a decrease of 1% will be coded -100.

For each economic measure, except the policy rate, we computed the total percentage of 2019 GDP spent by a given country over the period studied (variables with the prefix “*total_*”). Table 1 reports the descriptive statistics for the different measures at the country-day unit of observation.

**Table 1:** Descriptive statistics and definitions.

Variable	Mean	St.dev.	Min	Max
Fiscal	0.0096	0.2149	0	15.83
Wage support	0.0017	0.0563	0	6.3
Cash transfers	0.0001	0.0375	0	4.6
In-kind transfers	0.0002	0.0108	0	1.27
Tax cuts	0.0006	0.0284	0	3.85
Tax deferrals	0.0015	0.0801	0	13.8
Sectorial support	0.0027	0.0760	0	7.02
Credit schemes	0.0006	0.0323	0	4.7
Off budget	0.0051	0.2083	0	28.66
Policy rate	-0.1161	11.17	-2000	2000

Note: the statistics are computed for each country-day of the sample.

**Fig. 1:**
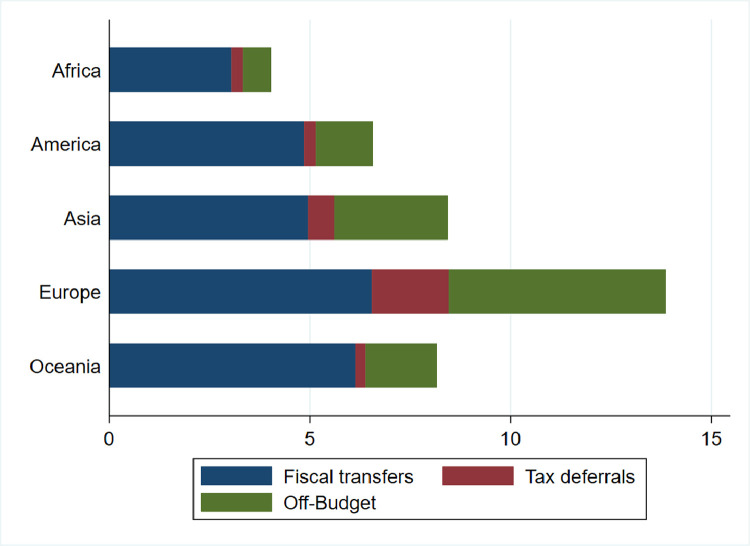
Fiscal measures, tax deferrals, and off-budget measures by continent. Note: The figure plots the average total percentage of GDP allocated to fiscal spending, tax deferrals, and off-budget measures in each continent as of June 1, 2021.

Fig. 1 shows the percentage of GDP allocated to fiscal measures, tax deferrals, and off-budget spending, aggregated by continent, as of June 1, 2021. European countries are by far the ones who most often use off-budget measures to counteract the effects of COVID-19 on the economy. This shows that European countries tend to implement indirect measures – such as credit guarantees or capital injections in companies – rather than more direct measures targeted at individuals or firms such as wage compensation.

**Fig. 2:**
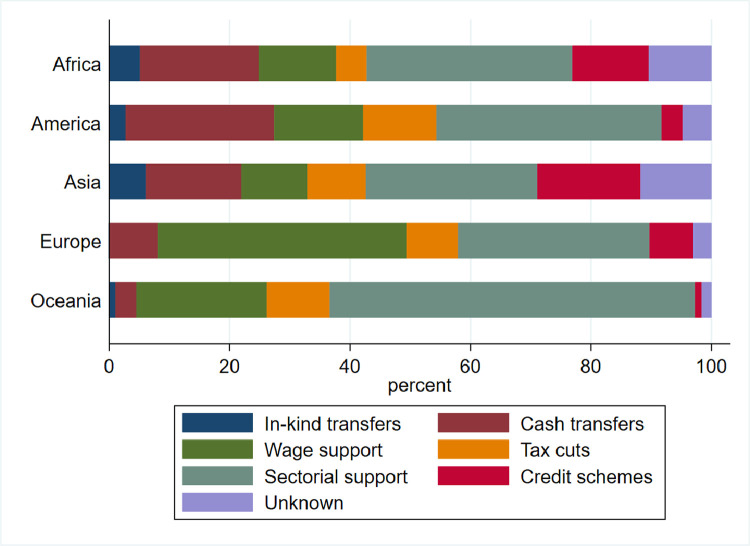
Distribution of fiscal spending by continent. Note: The figure plots the breakdown of fiscal measures in each continent as of June 1, 2021 for 101 countries for which the unknown part of the breakdown is inferior to 1 point of GDP in absolute value.

Fig. 2 shows the breakdown of fiscal spending for each continent regarding measures that are neither off-budget nor deferred, as of June 2021. Interestingly, in-kind transfers have been relatively more used in Africa and Asia. Cash transfers also represent a high share of the total spending in Africa, Asia, and America (including South America). These two observations are consistent with the idea that these regions mostly consist of developing countries, in which much of the economy is still informal. Europe is characterized by wider wage support while Oceania has a strong sectoral support (e.g. investments or increased budget for some sectors) due to its relative isolation.

**Fig. 3:**
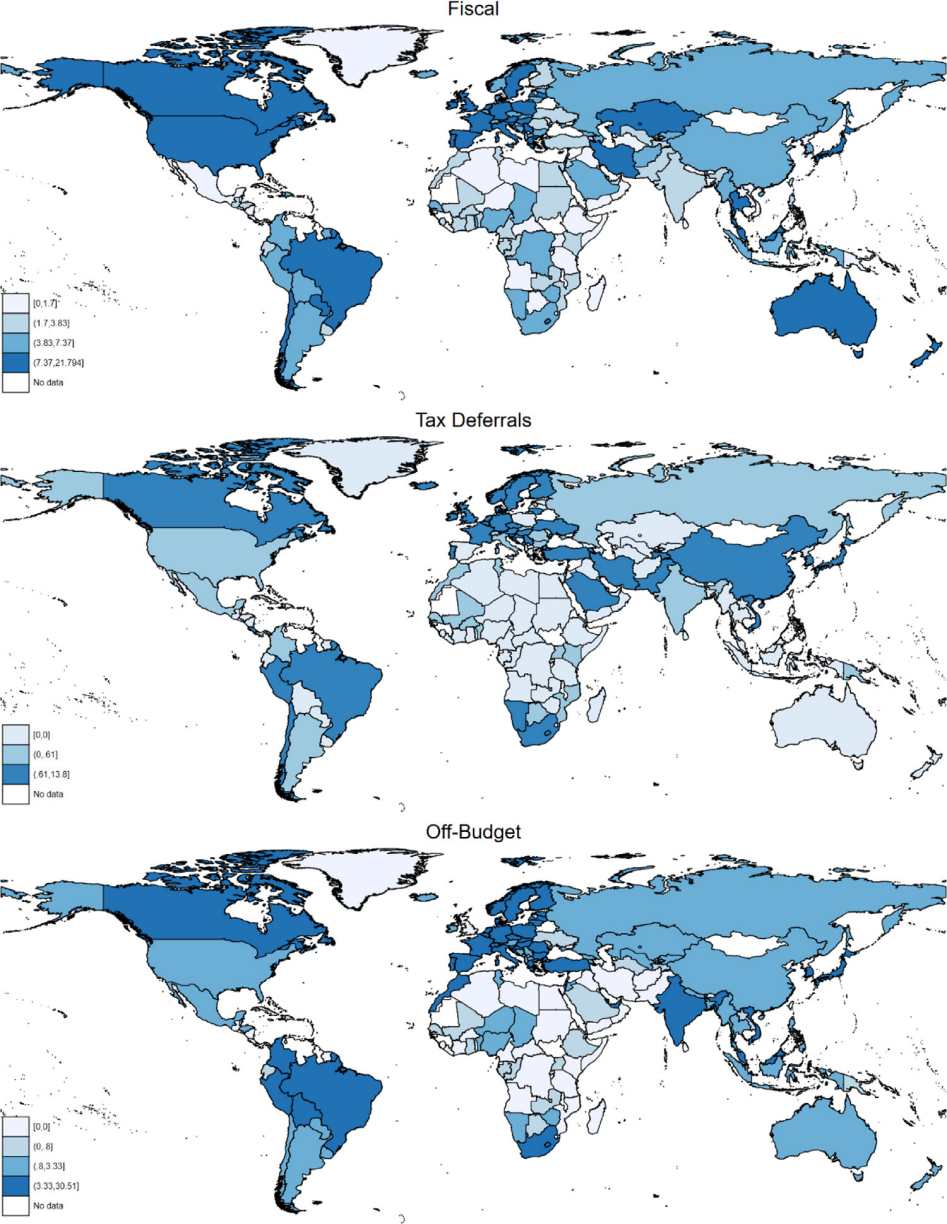
World map of fiscal measures, tax deferrals, and off-budget measures. Note: The maps represent the levels of spending for different measures (fiscal, tax deferrals, off-budget) at the country-level, as of June 1, 2021. Darker colors indicate higher spending.

**Fig. 4:**
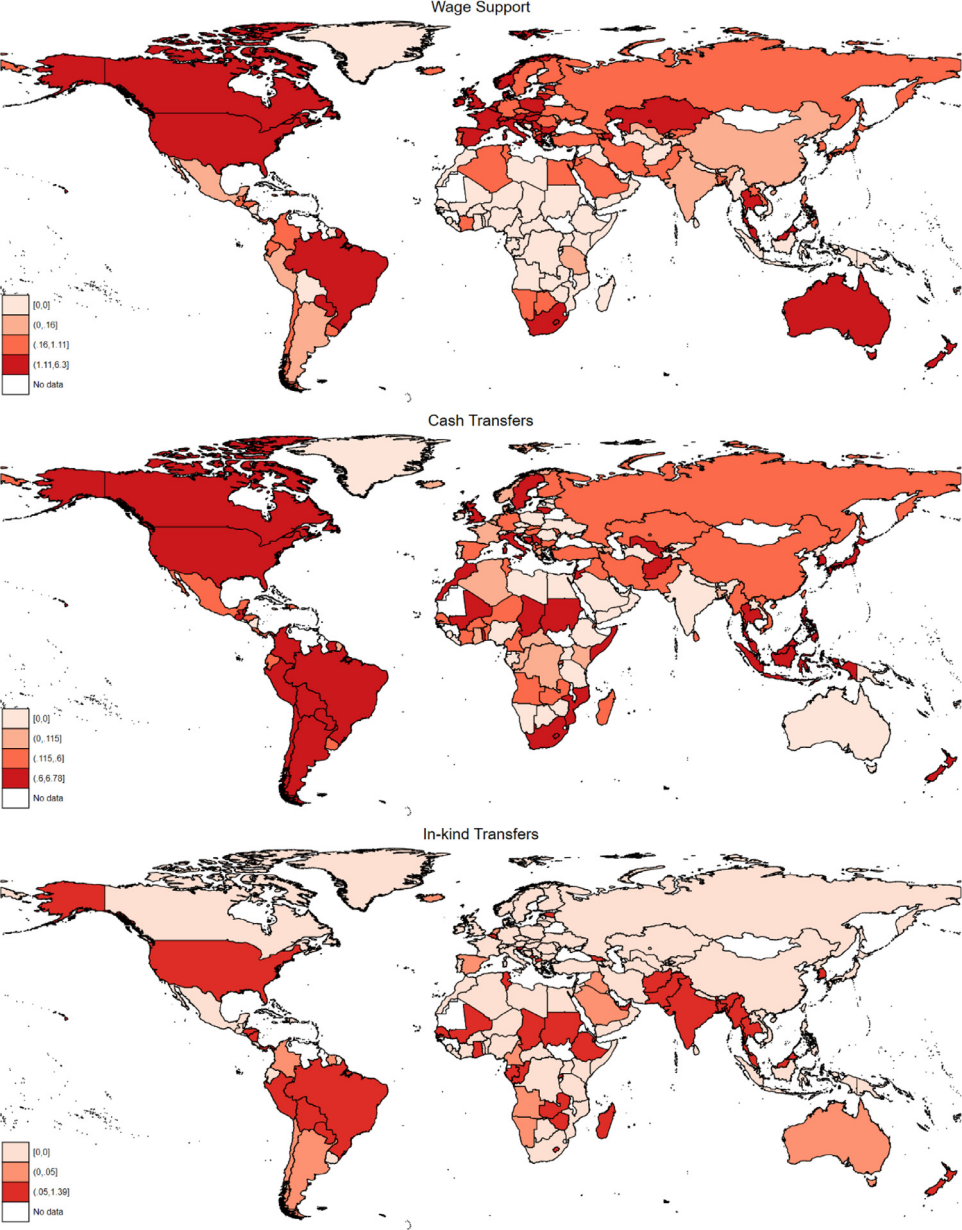

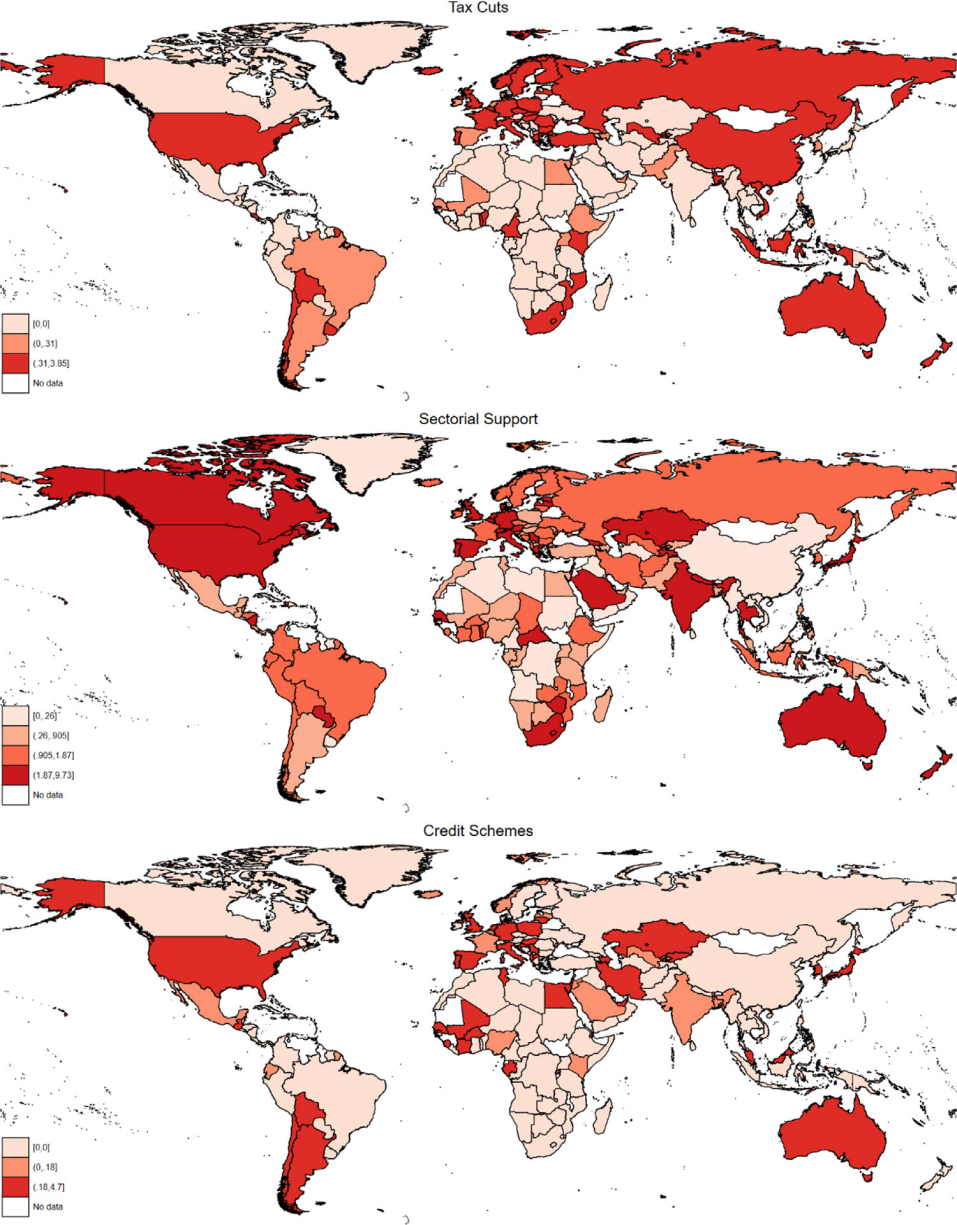
World map of the different fiscal measures. Note: The maps represent the levels of spending for different fiscal measures at the country-level. Darker colors indicate higher spending.

Figs. 3 and 4 map the intensity of measures at the country level, using world coordinates provided by Natural Earth.^2^ Fig. 3 maps the intensity of fiscal measures, tax deferrals, and off-budget measures. Darker colors indicate higher spending in a given measure. Fiscal measures are particularly important in industrialized countries, e.g., Europe and the United States. Tax deferrals and off-budget measures are also particularly important in European countries. Fig. 4 displays the intensity of the different fiscal measures at the country-level. Wage support schemes, tax cuts, and sectorial support were particularly generous in industrialized countries. Cash transfers are relatively more important in the Americas, but also in some Sub-Saharan and East Asian countries. In-kind transfers are usually more important in developing countries, e.g. South Asian, South American, and Sub-Saharan countries.

## 3 Experimental Design, Materials and Methods

### 3.1 Coding

For all variables, the coding of the data is done directly in a Stata do file via lines of coding, following the coding method developed by Porcher [3]. Each line of coding identifies the measure to be coded, the scale of the measure in percentage of GDP, the targeted country and the period for which the measure is implemented. The format of the coding is standard and easy to read, even by non-Stata users.

For example, the first plan for wage support was implemented in France on March 20^th^, 2020, for a global amount representing 1.18% of the French 2019 GDP, but only 0.27% were disbursed in the first round, while 0.91% were spent in the second fiscal plan. The line coded is *replace wage=1.18-0.91 if iso=="FRA" & tin(20mar2020, 20mar2020)*. The second fiscal plan implemented on April 15, 2020 activates the remaining wage support from the first plan (0.91%) and adds a bonus for health workers (0.03% of GDP). We thus coded *replace wage=0.91+0.03 if iso=="FRA" & tin(15apr2020, 15apr2020).*

The spreadsheet data are created from the coding script, available on the online repository of the dataset (“script_fiscal.do”). As noted by Porcher [3], there are many advantages to using such a methodology rather than writing directly in the spreadsheet:

1. The script can be run easily, and potential mistakes in the coding can be easily corrected: if the script does not write, or if it writes more than a unit when it is run, then this indicates a mistake in the coding.
2. Potential errors on measures, scales, dates and countries are more visible to the cross-checkers than they are in a spreadsheet format and can be directly corrected. Verifications or errors can be easily flagged.
3. Our coding scheme forces coders to search and fulfill the dates of implementation of the policies. It also provides more valuable knowledge of the dataset, as we can observe the changes in policies across economic stimuli implemented to face COVID-19.

### 3.2 Technical validation

Three different coders worked from January 2021 until June 2021 on the coding. The coding script was cross-checked by each coder on several occasions and several different points in time. This systematic verification process ensures a homogeneous coding method and avoids mistakes, especially those linked to contradictory interpretations of the classification of the different measures. It also allows, when necessary, correction of the script over time in cases where previously announced measures turned out to be misestimated or unimplemented.

We used the IMF Fiscal Monitor database and the IMF COVID-19 Policy Tracker to obtain information about the amounts spent. When necessary, we also used alternative sources to more precisely categorize the expenditures. All the extra sources that were used to feed the code are reported in an Excel file (‘Source.xls’), which is available on the repository of the dataset. This ensures transparent, impartial and consistent coding. These sources, however, had to be consistent with the information supplied by the IMF.

We are aware that collecting data from other sources might lead to the importation of any measurement issues they bring with them. However, we did our best to triangulate the information from the IMF with other sources.

### 3.3 Usage note

The dataset is based on the manual recording of economic measures implemented all around the world. We made our best attempt to capture the real nature of government spending to fight COVID-19, but there might be some remaining errors. Please email the corresponding author if you wish to point out any errors, or leave a message on the GitHub repository.

## Ethics Statements

This work meets the Elsevier ethical publishing requirements. This work does not involve studies with animals and humans, nor data collected from social media platforms.

## CRediT Author Statement

**Simon Porcher:** Conceptualization, Methodology, Data curation, Visualization, Investigation, Writing- Original draft preparation, Editing, Reviewing.

## Declaration of Competing Interest

The authors declare that they have no known competing financial interests or personal relationships that could have appeared to influence the work reported in this paper.

## Data Availability

Econ-Response2covid19 (Original data) (GitHub).

## Appendix Supplementary Materials

Image, application 1

## Footnotes

1 COVID-19 (Coronavirus): ADB's Response | Asian Development Bank COVID-19 | Economic Commission for Latin America and the Caribbean (cepal.org)

2 Natural Earth » 1:110m Cultural Vectors - Free vector and raster map data at 1:10m, 1:50m, and 1:110m scales (naturalearthdata.com)
